# Impact of children's migration on health and health care-seeking behavior of elderly left behind

**DOI:** 10.1186/1471-2458-11-143

**Published:** 2011-03-02

**Authors:** Ramesh Adhikari, Aree Jampaklay, Aphichat Chamratrithirong

**Affiliations:** 1 Geography and Population Department, Mahendra Ratna Campus, Tribhuvan University, Kathmandu, Nepal; 2 Institute for Population and Social Research, Mahidol University, Salaya, Phutthamonthon, Nakhon Pathom 73170, Thailand

## Abstract

**Background:**

Many countries are facing the burden of accelerated population aging and a lack of institutional support to meet the needs of older individuals. In developing countries, adult children are primarily responsible for the care of their elderly parents. However, out-migration of adult children is common in these countries. This study aims to explore the impact of migration on the health of the elderly left behind and their health care-seeking behavior.

**Methods:**

This paper uses data from a national survey of older persons in Thailand conducted in 2007. The analysis is confined to those who were aged 60 years or above and who had at least one child (biological or step/adopted) (n = 28,677). Logistic regression was used to assess the net effect of migration of adult children on the health of the elderly left behind and their health care-seeking behavior, after controlling for other socio-demographic and economic variables.

**Results:**

More than two-thirds of the elderly (67%) had at least one migrant child. About three-fifths (58%) reported that they had at least one symptom of poor mental health. Almost three in five elderly (56%) rated their health as poor, and 44% had experienced at least one chronic disease. About two-thirds of the elderly (65%) got sick during the 5 years preceding the survey. An overwhelming majority of elderly (88%) who got sick during the five years preceding the survey had sought treatment for their last illness.

After controlling for socio-demographic and economic variables, our study found that those elderly who had a migrant child were more likely (OR = 1.10; 95% CI 1.05-1.17) to have symptoms of poor mental health than those whose children had not migrated. However, no significant association was observed among physical health, such as experience of chronic disease, perceived poor health, and illness of the elderly left behind. Interestingly, however, out-migration of adult children was independently associated with higher utilization of health services. The elderly who had migrant children were more likely (odds ratio = 1.22, CI 1.11-1.33) than those whose children had not migrated to seek treatment for their most recent illness, after controlling for socio-demographic and economic variables.

**Conclusion:**

Our study provides novel evidence on an issue of special importance to countries affected by heavy out-migration of adult children, an issue that has received little attention. Out-migration of adult children was highly associated with poor mental health but it was not associated with the physical health of the elderly left behind. Out-migration of children was also highly associated with higher utilization of health facilities by the elderly. Thus, in order to decrease morbidity among the elderly as well as to maintain and enhance the well-being of families, programs should focus on alleviating the symptoms of poor mental health among the elderly left behind and aim to reduce the differences in utilization of health care-seeking behavior among elderly with children present in the community and elderly left behind.

## Background

In modern times, many factors have intensified migration. Improvement in transportation infrastructure, advances in communications technology (which makes keeping in contact with left-behind over much easier and cheaper), increased trade between countries (including the impact of trade resulting from globalization), political instability, poverty, and unemployment in economically disadvantaged areas/countries are some of the important factors that contribute to this phenomenon [1].

The interaction between health and migration is complex and dynamic. Migration can have an impact on physical, mental, and emotional health and well-being of migrants themselves, on those left behind in the place of origin, and on those at the destination [2]. The connection between health status and migration is clearly bidirectional. In one direction, the act of migration may influence health outcomes. In the other direction, a person's health may influence one's propensity to migrate or the destination one chooses [3].

In many places, and especially in most developing countries, the growing proportion of aging individuals challenges public and social institutions responsible for elderly care. These countries face an additional burden of accelerated population aging and a lack of institutional support to meet the needs of older individuals [4]. Changes in demographic events, especially sharp declines in fertility and mortality, have resulted in rising life expectancies and increasing rates of population aging [5]. As is the case in most other countries in Asia, the elderly in Thailand have traditionally relied on their children for personal care and financial support [6]. Trends in declining fertility and increasing internal migration have prevailed in Thailand since 1985, when a period of sustained economic development began [7,8]. These patterns have resulted in decreasing numbers of children available to care for their elderly parents. Increasing out-migration of young adults has created concerns about whether the absence of children in the household or community affects the health of elderly left behind or their health care-seeking behavior. Existing literature on the consequences of migration has focused mainly on the impact of migration on receiving areas and on migrants themselves but has paid little attention to the effects of migration on sending communities and of the family members "left behind" by migrants [9-11].

Evidence from the literature shows that migration can affect the health of those left behind both positively and negatively. With additional money coming from remittances, people have easier access to health services, can buy expensive medicine, and eat better quality food [12]. Thus, migration can benefit both migrants and left-behind family members as it would enhance their well-being [13,14]. Remittances received from migration could support the family left behind by minimizing economic risk and overcoming capital constraints [14,15]. A study in South Africa found that temporary internal migrants, by increasing their household income, were able to bring positive health outcomes not only for themselves but also for family members [16] including elderly parents left behind [17]. Other research also shows that migration can lead to better health among the population left behind [18-20]. Similarly, a study conducted by Abas et al. [21] found that out-migration of children was independently associated with less depression in parents.

On the other hand, some research notes that out-migration of young adults has severe negative consequences for ageing parents, namely, loneliness, isolation, and the loss of basic instrumental and economic support [22]. A study of Mexico-US migration found evidence of a causal link between poor elderly health outcomes and children's migration to the US [23]. Research in Bangladesh has found that those left behind by adult migrant children face risks stemming from the loss of personal support and care [18].

Although the separation of families due to migration might have serious implications on the health status of elderly left behind and their health care-seeking behavior, the possible health effects on elderly left behind in Thailand remain unclear. This study aims to explore the impact of migration of adult children on the health of the elderly left behind and on their health care-seeking behavior. In-depth examination of these issues will provide policy makers and program planners with needed information and will raise public attention to this public health issue. The findings of this study will help fill the gap in the literature and provide the understanding necessary for advocacy and for the design of appropriate interventions related to migration in Thailand.

## Methods

### Sources of data and study design

This paper uses data from a cross-sectional survey conducted in 2007 among the elderly in Thailand. This nationally representative survey was conducted by the National Statistical Office (NSO) of Thailand. The NSO has conducted three nationally representative household surveys of older persons, in 1994, 2002, and 2007 [24-26] to fulfill the need for adequate information to develop appropriate policies and programs to ensure the well-being of the Thai elderly. These surveys collected information on socioeconomic conditions, living arrangements, income, health status, and health care-seeking behavior of the elderly in Thai society

The survey is based on a probability sample of persons aged 50 years old or older who were usual residents of their own or family members' households. A stratified two-stage sampling procedure was employed to collect the information. The primary sampling units were blocks for municipal areas and villages for non-municipal areas. The secondary sampling units were households, using random sampling from the list of all enumerated households in each block or village selected. The number of households selected was 15 households per block in municipal areas and 12 households in non-municipal areas. In total, 56,002 persons were interviewed. However, the analysis for this paper is confined to those who were aged 60 years or above and who had at least one child (biological or step/adopted) (n = 28,677). A structured questionnaire was used by trained interviewers to interview selected subjects at the household level. This study was approved by the ethics committee of the NSO of Thailand.

### Dependent and independent variables

#### Dependent variables

This study examines several aspects of the health of elderly parents. These include symptoms of poor mental health, self-assessed health status, chronic diseases, illnesses, and treatment-seeking behavior.

##### Symptoms of poor mental health

Items measuring symptoms of poor mental health experienced by elderly persons during the month preceding the survey were: i) feeling stress, ii) feeling moody, iii) feeling hopeless, iv) feeling useless, v) feeling down/unhappy, and vi) feeling lonely. One composite indicator, "symptoms of poor mental health," was developed from all the above-listed symptoms (Cronbach's α = 0.86), resulting in 4 categories: had no symptoms of poor mental health, had 1 symptom, had 2 symptoms, and had 3 or more symptoms. For the logistic regression, two further categories were created: "no symptoms poor mental health" and "at least one symptom of poor mental health".

##### Self-assessed health

Respondents in the survey were asked, "How has your health been in the past 7 days?" The question had five response categories (very good, good, fair, bad, and very bad). The authors categorized the response categories into two groups: "good," which included "very good" and "good," and "poor," which included "fair," "bad," and "very bad."

##### Chronic diseases

Individuals were asked about the presence of hypertension, heart disease, diabetes, cancer, stroke, and paralysis. One composite indicator, "chronic disease condition," was developed from all the chronic diseases, resulting in 4 categories: had no chronic disease, had 1 chronic disease, had 2 chronic diseases, and had 3 or more chronic diseases. For the logistic regression, two more categories were created: "no chronic disease" and "at least one chronic disease."

##### Illness

The study explored whether the elderly had symptoms of an illness (self-reported) during the five years preceding the survey. This variable was split into two categories: "experienced symptoms" and "did not experience symptoms."

##### Treatment sought

The study explored whether the elderly who experienced symptoms of an illness during the five years preceding the survey sought treatment for the most recent illness. This variable was categorized into two categories: "sought treatment" and "did not seek treatment."

#### Independent variables

##### Migration of adult children

In this study, we have defined an out-migrant child as one living outside their parents' province. We have used migration of adult children as a main independent variable. This variable was split into two categories: "0 no migrant child" and "1 at least one migrant child."

The other demographic and socioeconomic variables were used as control variables in this study. Many previous studies have shown that demographic and socioeconomic variables have strong effects on health and health care-seeking behavior. Hence we needed to control for their effect to determine the independent effect of migration. Demographic variables included age (60-69 years, 70-79 years, and 80 years and above), sex (male and female), place of residence (urban and rural), and number of respondent's children. The economic variable was the average total income per year (100,000 baht or more, 30,000-99,999 baht, 10,000-29,999 baht, and less than 10,000 baht). Social variables included education (higher than secondary level, secondary level, primary/elementary level, no schooling) and living arrangements (living alone, living with children, living with other relatives).

### Methods of analysis

Analysis for this study was confined to those who were aged 60 years or above and who had at least one child (biological or step/adopted) (n = 28,677). Univariate and multivariate analysis were used to analyze the data. Initially, univariate or descriptive analysis was used to describe the respondents' socio-demographic characteristics. Then, after controlling for the other socio-demographic and economic variables, multivariate analysis in the form of logistic regression was used to identify whether migration of adult children affected the elderly's likelihood of experiencing symptoms of diseases, self-assessed health, experience of chronic diseases, illnesses, and treatment-seeking behavior.

## Results

### Sociodemographic and economic characteristics of the sample population

More than half of the sampled respondents were aged 60-69 while about one out of seven were aged 80 or above, and nearly three out of five were female. The large majority had only an elementary-level education. Respondents had an average of 4 children. About three in five respondents were from rural areas. Half reported that their household income per year was about 30,000 baht or less. Only about one in ten elderly lived alone, and two-thirds had at least one migrant child. About four in five elderly (79%) who had at least one migrant child had received money from their child(ren) during the 12 months preceding the survey (Table 1).

**Table 1:** Selected background characteristics of the sampled elderly

Characteristics		%	N
**Age group**	60-69 years	52.6	15,089
	70-79 years	34.3	9,824
	80 years or +	13.1	3,764

**Sex**	Male	44.0	12,626
	Female	56.0	16,051

**Place of residence**	Rural	57.1	16,365
	Urban	42.9	12,312

**Level of education**	Higher than secondary	3.8	1,080
	Secondary level	7.1	2,026
	Primary or below	89.2	25,571

**Number of children**	One	8.0	2,303
	Two	16.3	4,688
	Three	19.7	5,653
	Four	17.5	5,030
	Five or more	38.4	11,003
	***Mean no. children***	**4.2**	

**Living arrangement**	Alone	7.5	2,151
	With child (ren)	61.9	17,742
	With other relatives	30.6	8,784

**Average household income per year**	100,000 baht or more	16.0	4,578
	30,000-99,999 baht	33.9	9,715
	Less than 30,000 baht	50.1	14,363

**Migration of child(ren)**	No	32.8	9,402
	Yes	67.2	19,275

**Total**		100.0	28,677

**Received money (baht) during the preceding 12 months from migrant children#**	None	20.9	4,031
	Up to 4999 baht	21.8	4,199
	5000-9999 baht	17.8	3,424
	10000-29999 baht	25.6	4,930
	30000 or above baht	14.0	2,691

**Total**		100.0	19,275

# Only those elderly who had migrant child(ren), 1USD = approx. 30 Baht

### Health status and health care-seeking behavior

Table 2 shows that a slightly higher proportion of elderly who had migrant child(ren) had at least one symptom of poor mental health during the month before the survey compared to those whose children had not migrated (56%). Furthermore, more than half (56%) of the elderly rated their health as poor, and nearly half (44%) reported that they suffered from at least one chronic disease. Overall, about two-thirds of the elderly had symptoms of an illness (65%) during the five years preceding the survey. No observed differences were found among elderly (by migration status of children) in terms of self-assessed health, number of chronic diseases, or illnesses during the previous 5 years.

**Table 2:** Health status and healthcare-seeking behavior among elderly according to migration status of child(ren)

	With migrant child(ren)	Without migrant child	Total
**Symptoms of poor mental health**			
No symptom	41.0	44.1	42.0
1 symptom	14.3	13.3	14.0
2 symptoms	16.1	15.4	15.9
3 or more symptoms	28.5	27.3	28.1

**Self assessed health**			
Good health	43.8	44.5	44.0
Poor health	56.2	55.5	56.0

**Number of chronic diseases**			
No	55.9	56.2	56.0
1 disease	29.1	29.2	29.1
2 diseases	11.7	11.6	11.7
3 or more diseases	3.3	3.0	3.2

**Illness during the previous 5 years**			
Yes	64.6	64.7	64.7
No	35.4	35.3	35.3

**Total**	**100.0**	**100.0**	**100.0**

**N**	**19,275**	**9,402**	**28,677**

**Treatment sought for the most recent illness+**			
Yes	89.0	86.5	88.2
No	11.0	13.5	11.8

**Total**	**100.0**	**100.0**	**100.0**

**N**	**12,455**	**6,086**	**18,541**

Note: + = only those who felt sick during the previous 5 years

It is encouraging to note that an overwhelming majority of elderly who felt sick (88%) had sought treatment for their most recent illness. Interestingly, a higher proportion of the elderly who had migrant child(ren) had sought treatment for the last illness compared to those whose children did not migrate (Table 2).

### Multivariate analysis

Logistic regression was used to assess the net effect of the migration of children on the health of the elderly left behind and their treatment-seeking behavior after controlling for the other control variables in the model.

Separate logistic regressions were run for 5 dependent variables. The odds ratio is presented in Table 3. Control variables used in this logistic analysis were categorical indicators of age (60-69, 70-79, and 80 or above), sex (male, female), place of residence (rural, urban), level of education (higher than secondary, secondary, and primary and below), living arrangements (living alone, with children, with other relatives), number of children respondents had, and average household income per year (100,000 baht or more, 30,000-99,999 baht, and less than 30,000). In the model, the reference group of the independent variable is "no migration of adult children."

**Table 3:** Odds ratio (OR) and 95% confidence interval (CI) for the effect of children's out-migration on elderly's experience of health problems (symptoms/diseases), perceived health status, chronic disease, illness and treatment-seeking behavior

	No out-migration of adult children = reference category		
**Dependent variables**	**OR**	**CI**	**Cox & Shell R square**

**Mental health status (n = 28, 677)**			

**Symptoms of poor mental health **(ref: No symptom)			
At least one poor mental health symptom	1.10***	1.05-1.17	0.124

**Perceived health status within previous 7 days (n = 28, 677)**			

**Self-assessed health **(ref: Good )			
Poor	1.03	0.98-1.09	0.128

**Chronic diseases (n = 28, 677)**			

**Chronic disease **(ref: No)			
At least one chronic disease	1.02	0.97-1.08	0.161

**Illness (n = 28, 677)**			

**Felt sick during the previous 5 years **(ref: No)			
Yes	1.00	0.95-1.07	0.116

**Healthcare-seeking behavior (n = 18, 541)**			

**Treatment sought for the last illness **(ref: No)			
Yes	1.22***	1.11-1.33	0.113

*Notes: Logistic regression was run separately for each dependent variable*.

*Models were fitted after controlling for age, sex, place of residence, education, number of children, living arrangements, and household income*.

*** = p < 0.001

Migration of children was found to have a strong association with poor mental health symptoms among the elderly left behind. Those elderly who had at least one migrant child were more likely (OR = 1.10; 95% CI 1.05-1.17) to have poor mental health symptoms than those whose children had not migrated. On the other hand, no significant association was observed with physical health, such as experience of chronic disease, self-rated health status, or experience of symptoms of illness during the previous 5 years.

Furthermore, the results show that the migration of children is a significant predictor of treatment-seeking behavior among the elderly left behind. For instance, those elderly who had at least one migrant child were more likely (OR = 1.22; 95% CI 1.11-1.33) to seek treatment for their most recent illness than were those who had no migrant children (Table 3).

## Discussion

This study has attempted to investigate the impact of out-migration of adult children on health issues and health care-seeking behavior of the elderly left behind in a society in which, traditionally, children take responsibility for older parents. Findings show that the majority of the elderly (68%) have at least one out-migrant child and that an overwhelming majority (79%) received money from their migrant children.

Out-migration of adult children was highly associated with poor mental health symptoms among the elderly left behind. It is likely that out-migration of children reduces opportunities for face-to-face interaction. This finding is similar to other studies [22,23] that found that out-migration of young people increased the loneliness felt by their aging parents. However, the findings of present study conflict with those of Abas et al., which found that out-migration of children was associated with less depression in parents [18]. This could be due to the fact that in their study most of the parents with migrant children were male, better educated, and married which would reduce the risk of depression.

Our study found that the migration status of children was not associated with the physical health of the elderly left behind (as measured by self-rated health, experience of chronic diseases, and illnesses during the 5 years preceding the survey). It should be noted that this may be because adult children are less likely to migrate when a parent is ill [27]. However, out-migration of adult children was independently associated with higher utilization of health facilities among the elderly left behind. It is encouraging to note that a significantly higher proportion of the elderly who had migrant children sought treatment compared to those who had no migrant child. This may be a result of remittances from migrant children, which make healthcare affordable. Indeed, about four-fifths of the sampled elderly had received money from their migrant children in the year preceding the survey.

An earlier national survey in Thailand also found that 77% of Thai elderly reported receiving financial support from children during the previous year [28]. Furthermore, after controlling the demographic, and socio-economic variables ^a^, we also found that those who had received remittances from their children were more likely (OR = 1.43, 95% CI 1.30-1.57) to seek treatment than those who had no migrant children. This could be due to the fact that the benefits of remittances offset the potential negative effect of migrant children living apart from their parents. The financial support provided by migrant children may operate primarily through amelioration of financial hardship of their parents. Studies have also found that most elderly Thai parents are getting financial support from their children and that this contributes positively to their material well-being [29,30]. Many families with migrant members moving within and between low- and middle-income countries tend to benefit economically [31] and to enjoy improved general health [20]. The other reason could be that adult migrant children can provide emotional support to their parents back home (by visiting home or via frequent telephone calls, mail, etc.) or provide both emotional and financial support [32] that help parents maintain good health through improvement of emotional and psychosocial well-being [33]. Yet another reason could be health knowledge transfers between migrants and their parents who are left behind.

There are some limitations in the interpretation of the results of this study. Because of the survey's cross-sectional design, all of the factors analyzed in the study were measured at a single point in time. Thus, the analysis can only provide evidence of statistical association between those items and the health status of the elderly left behind and health care-seeking behavior; it cannot show a cause-effect relationship. Besides, we have to be careful while comparing the results of this study with those of other research studies that have used pre-existing validated questionnaires to measure mental and physical health.

## Conclusion

Our study provides novel evidence on an issue of special importance to countries affected by heavy out-migration of adult children, an issue that has received little attention. Findings here show that out-migration of adult children is highly associated with poor mental health but is not associated with the physical health of elderly left behind. Out-migration of children is also highly associated with greater utilization of health facilities. Thus, in order to decrease morbidity among the elderly as well as to maintain and enhance the well-being of families, programs should focus on alleviating the symptoms of poor mental health among the elderly left behind and aim to reduce the differences in utilization of health care-seeking behavior among elderly with children present in the community and elderly left behind.

## Endnotes

**a. **We were unable to keep both 'remittances' and 'out migration of children' in the same model as it is highly correlated. Therefore, we run separately to see the association between 'remittance' and 'treatment seeking' variables after controlling for other demographic and socio-economic variables.

## Competing interests

The authors declare that they have no competing interests.

## Authors' contributions

RA analyzed, interpreted the data, and drafted the manuscript. AJ and AC commented and provided suggestions on the analysis and interpretation. All authors read and approved the final version of the manuscript.

## Pre-publication history

The pre-publication history for this paper can be accessed here:

http://www.biomedcentral.com/1471-2458/11/143/prepub
